# Genetic risk variants in New Yorkers of Puerto Rican and Dominican Republic heritage with Parkinson’s disease

**DOI:** 10.1038/s41531-023-00599-6

**Published:** 2023-12-07

**Authors:** Gabriel Miltenberger-Miltenyi, Roberto A. Ortega, Aloysius Domingo, Rachita Yadav, Ayumi Nishiyama, Deborah Raymond, Viktoriya Katsnelson, Nikita Urval, Matthew Swan, Vicki Shanker, Joan Miravite, Ruth H. Walker, Susan B. Bressman, Laurie J. Ozelius, José C. Cabassa, Rachel Saunders-Pullman

**Affiliations:** 1 Laboratório de Genética, Faculdade de Medicina, Universidade de Lisboa, Lisbon, Portugal; 2 Department of Neurology, Ludwig-Maximilians-Universität München, Munich, Germany; 3 Department of Neurology, Icahn School of Medicine, Mount Sinai, New York, NY USA; 4 Department of Neurology, Mount Sinai Beth Israel, New York, NY USA; 5 Department of Neurology, Massachusetts General Hospital, Boston, MA USA; 6 Center for Genomic Medicine, Massachusetts General Hospital, Boston, MA USA; 7 Program in Medical and Population Genomics, Broad Institute, Cambridge, MA USA; 8 Department of Neurology, James J. Peters Veterans Affairs Medical Center, Bronx, NY USA

**Keywords:** Parkinson's disease, Risk factors

## Abstract

There is a paucity of genetic characterization in people with Parkinson’s disease (PD) of Latino and Afro-Caribbean descent. Screening *LRRK2* and *GBA* variants in 32 New Yorkers of Puerto Rican ethnicity with PD and in 119 non-Hispanic-non-Jewish European PD cases revealed that Puerto Rican participants were more likely to harbor the LRRK2-p.G2019S variant (15.6% vs. 4.2%, respectively). Additionally, whole exome sequencing of twelve Puerto Rican and Dominican PD participants was performed as an exploratory study.

With the advent of personalized medicine emerging in the neurology landscape, genetic testing is playing a greater role in counseling individual patients with Parkinson’s disease (PD). *LRRK2* and *GBA* are the most prevalent genetic risk factors for PD^1,2^. Mutations in both *LRRK2* and *GBA* occur worldwide, and founder effects have been described in some populations^3,4^. *LRRK2* and *GBA* variant carriers with PD can be enrolled in neuroprotection trials^5,6^; knowledge of genetic status is required to determine trial eligibility^7,8^. Thus, study recruitment may be biased toward better studied populations with known higher mutation frequency^9,10^. Despite suggestion that *LRRK2* variants may be increased in Puerto Ricans^10,11^, mutation rates in individuals with self-reported Puerto Rican ethnicity^10,12,13^ have not been widely studied.

Understanding the frequencies of potential variants as genetic risk in different populations is especially important as genetic testing shifts to whole -exome and whole -genome sequencing (WES and WGS) whose interpretation rests on these data. However, the majority of exomes in population databases, such as the Genome Aggregation Database (gnomAD v2, https://gnomad.broadinstitute.org/), are from mixed European Caucasians (45% of the >125,000 exomes in gnomAD) while Latino and African origin populations are still underrepresented.

We analyzed the frequencies of the LRRK2-p.G2019S and specific *GBA* variants in a group of PD participants with self-reported Puerto Rican ethnicity, and also carried out an exploratory WES analysis in a subgroup of Puerto Rican participants as well as a limited number of Dominican PD participants.

Among 32 PD participants with self-reported Puerto Rican ethnicity, and 119 with non-Hispanic, non-Jewish European ancestry, those from Puerto Rico were more likely to harbor the LRRK2-p.G2019S variant than Europeans [Table 1A; 15.6% vs. 4.2%) (OR 4.22 (95% CI: 1.14–15.6)].

**Table 1:** Clinical characteristics: (A) Participants for the LRRK2-p.G2019S and *GBA* variant screening. (B) Participants selected for WES (from Mount Sinai Beth Israel).

(A) Participants for the LRRK2-p.G2019S and *GBA* variant screening				
Characteristics		Self-reported ethnicity		*P*
		Puerto Rican	Non-Hispanic European	
Overall: *N*		32	119	
Sex: N women (%) total		15 (46.9%)	51 (42.9%)	0.684
Age at PD onset (years, mean ± SD)		53.3 ± 15.0	54.7 ± 10.9	0.748
LRRK2-p.G2019S carriers: *N* (%)		5 (15.6%)	5 (4.2%)	0.031*
Sex: *N* women (%) LRRK2 carriers		1 (20%)	3 (60%)	0.197
Age at PD onset (years, mean ± SD)		53.0 ± 8.0	59.2 ± 4.1	0.222
*GBA* variant carriers: *N* (%) p.N370S p.R496H p.L444P RecNcil IVS2 + 1 p.E326K p.T369M		0	14 (13.0%) 2 (2 F, 0 M) 1 (1 F, 0 M) 2 (1 F, 1 M) 1 (0 F, 1 M) 0 4 (4 F, 0 M) 4 (1 F, 3 M)	0.131
Age at PD onset (years, mean ± SD)		–	53.9 ± 8.9	–
Dual LRRK2-p.G2019S/GBA-p.T369M variant carrier		1	0	–
Age PD onset (years, mean ± SD)		62	–	–
(B) Patients selected for WES				
ID	Sex	Age of onset	Family history (inheritance)	Country of Origin
1	F	61	Yes (AD)	DR
2	F	45	Yes (AD)	DR
4	M	38	No	PR
5	M	46	No	DR
6	F	24	Yes (unclear)	PR
7	F	37	Unknown	PR
8	F	58	Yes (unclear)	PR
9	M	57	Unknown	PR
10*	F	17	No	PR
11	F	41	No	DR
12	M	41	Yes (unclear)	PR
13	F	47	No	PR

(A) LRRK2-p.G2019S and *GBA* variant status, sex and age at PD onset available in 30/32 and 29/32 participants self-reporting Puerto Rican ancestry, resp. and in 115/119 and 113/119 self-reporting European ancestry, respectively, *OR (95% CI): 4.22 (1.14, 15.6). One Puerto Rican participant carried both the LRRK2-p.G2019S and the *GBA*-p.T369M variations.

(B) *PR* Puerto Rico, *DR* Dominican Republic, *F* female, *M* male, *AD* autosomal dominant; *WES was performed, but the participant was excluded from the comparisons of AF in cases and controls, due to report of pathogenic PRKN deletions: (1) del Exon 3, and (2) del Exons 2–4.

Fourteen European participants carried a *GBA* variant while no Puerto Rican cases had an isolated *GBA* variant; one carried dual *LRRK2-GBA* variants (Table 1A).

Additionally, because so few Latino PD patients have been examined for known PD candidate genes at present, as a pilot study, 8 patients of Puerto Rican ethnicity from the initial screening and 4 further patients of Dominican Republic (DR) ethnicity with early age of onset and/or positive family history (FH) underwent WES (as the most readily available tool for detecting potentially relevant coding variants) and standard variant discovery analyses (Table 1B). A search for rare (gnomAD overall allele frequency [AF] <0.01) deleterious variants in 74 disease-associated genes (Supplementary Table 2) identified variants in 8 genes (Table 2), including one in *TREM2* (rs2234253) in patients 2 and 5 (Table 1B) both of DR ethnicity with tremor predominant PD; and one in *CTSB* (rs28605689) in patient 6 (Table 1B) with postural instability and gait disorder (PIGD). Neither of these variants were reported in ClinVar (https://www.ncbi.nlm.nih.gov/clinvar/).

**Table 2:** Rare deleterious variants detected from exomes of Caribbean Latino PD patients in New York.

Gene	Variant	Allele	cDNA pos	CADD Phred	SIFT	PolyPhen	MACIE Score	No. in Hisp PD	gnomAD2.1 total AF	Latino AF	African AF	NFE AF	ClinVar	Patient (See Table 1B)
*BIN1*	rs112318500	A	2007	28.9	Del	pos dam	0.895954048	1	0.009842	0.004459	0.1017	0.0003486	5 (3 unknown phenotype, 2 myopathy)	2
*CSF1R*	rs34951517	T	1529	20.9	Tol	ben	0.860415109	1	0.009276	0.008725	0.003125	0.01424	5 (1 unknown phenotype, 1 leukoencephalopathy)	4
*DNAJC6*	rs78141380	T	1657	23.1	Del	ben	0.927191289	1	0.003653	0.001129	0.03882	0.00006197	4 (2 unknown phenotype, 2 juvenile PD)	1
*MAPT*	rs143956882	T	1602	28.5	Del	pro dam	0.957735057	1	0.001661	0.001919	0.0004005	0.002167	4 (2 unknown phenotype, 2 frontotemporal dementia)	1
*TREM2*	rs2234253	T	381	24.8	del	pro dam	0.947777545	2	0.01294	0.007845	0.1285	0.001006	not in ClinVar	2, 5
*CTSB*	rs28605689	G	331	26.9	del	pro dam	0.985887869	1	0.0004989	0.000113	0.005342	0.000007746	not in ClinVar	6
*BST1*	rs34163939	A	910	27.5	del	pro dam	0.891834553	3	0.007395	0.005378	0.07254	0.0003255	1 (unknown phenotype)	1, 5, 6
COBL (stop)*	7:51096464	A	2495	35	–	–	–	1	not in gnomAD				not in ClinVar	1
COBL**	rs202117145	A	3714	25	del	pos dam	0.925428514	1	0.00004643	0.0001977	0.00004073	0.00001576	not in ClinVar	8

Variants with a gnomAD overall allele frequency [AF] <0.01 are presented. SIFT: del deleterious, tol tolerated, Polyphen: pro dam probably damaging, ben benign, pos dam possibly damaging. Interpretation of CADD-Phred: variants with scores >30 are predicted to be in the 0.1% most deleterious possible substitutions.

*AF* allele frequency, *NFE* non-Finnish European.

*the stop-gain mutation in COBL was detected via a search for loss-of-function mutations genome-wide (in contrast to the first seven variants in the table, which were detected from a candidate-gene approach, see Methods).

**upon detecting the stop-gain mutation in COBL, we searched for other variants in this gene in our exome cohort, revealing this deleterious missense mutation in another sample. The gene COBL is moderately depleted for loss-of-function mutations in the population based on a LOUEF score of 0.47 (gnomad.broadinstitute.org).

To detect potential risk alleles for PD in the Puerto Rican/Dominican populations, variant AF in our sample was compared to AF in control populations (gnomAD overall, Latino, African, (Non-Finnish) European (NFE)) for variants in the candidate gene list. While this analysis did not reveal a significant enrichment in PD of any single variant, we noted a nominally increased frequency of rs2230570 (*EIF4G1*) as compared to the Latino population in gnomAD v2 (Supplementary Table 1; *p*-value 0.008; adjusted *p*-value 0.052). This variant showed a similar trend in the NFE population (*p*-value 0.023; adjusted *p*-value 0.131) but was not significant when compared to the African population (Supplementary Table 1). Although this variant is predicted to be benign, it could be in linkage disequilibrium with some other functional variant in non-coding region not assessed by WES.

Evaluating loss-of-function variants exome-wide, we discovered a novel stop-gain mutation in exon 10 (of 13) of the *COBL* gene (p.Glu777*) in patient 1 (Table 1B) who is affected with mild parkinsonism and positive FH. Follow-up analysis revealed a rare deleterious missense variant (rs202117145, Table 2) in the same gene in patient 8 (Table 1B) with gait onset and rest tremor. Neither variant is reported in ClinVar.

Studies on the genetics and clinical spectrum of PD in non-European, especially Latino and Afro-Caribbean, populations are limited and mainly focusing on one or a few PD- associated genes^14,15^. Such studies are sorely needed, as the demand for direct-to-consumer testing supports the patients´ interest in determining the genetic contribution to their PD. There is also emerging evidence that awareness of genotype could guide therapeutic decisions^7,8^. Trials for some genes have emerged and multiple are in progress, thus far focused on *LRRK2* and *GBA* variants^16,17^.

We have previously reported an excess of the LRRK2-p.G2019S variant^10^ in Puerto Ricans, however, the numbers of screened samples have been relatively small. Here, the increased frequency of LRRK2-p.G2019S in our New York Puerto Rican population was confirmed (15.6%), suggesting that testing in individuals of Puerto Rican ethnicity who are potentially eligible for trials should be considered as it will improve access and equity. However, Nuytemans et al. reported that only 2/37 individuals (5.4%) of Puerto Rican ethnicity ascertained from Manati and Miami harbored p.G2019S mutations^13^. Local ancestry analysis showed that these two patients had likely European ancestry, suggesting that p.G2019S was introduced to the Latino population through their European ancestor. Further studies of larger cohorts are thus still needed to obtain better frequency estimates. Lower frequencies of p.G2019 were also observed in the PD populations of Argentina (3.2%), Brazil (1.4%), Colombia (1.5%), Ecuador (1.2%), Peru (0.2%), and Uruguay (4.2%)^18^ and this raises important questions about why even in the Nuytemans study, the rates were slightly increased among Puerto Ricans and whether this is attributable to a greater frequency in the European than the Amerindian ancestry. As the Puerto Rican population represents an admixture of native Taino, African and Hispanic European, thus ancestry informative markers might further help guide such data. Admixtures of European, African, and Amerindian ancestries have similarly been reported in the Puerto Rican PD patients from Miami and Manatí^13^.

We carried out principal component analysis (PCA) for ancestry related markers for the 12 patients who were included in the WES section. This analysis showed that these patients have admixed genetic background with contributions from European, American (Hispanic) and African global populations (Supplementary Fig. 1). Whether the Puerto Rican ethnicity contains higher proportion of European (non-Ashkenazi) ancestry remains to be determined.

As other genetic variants may be contributing to PD in the Puerto Rican population, and with the advent of WES and WGS, it is important to extend beyond LRRK2-p.G2019S and more frequently assessed *GBA* variants, and to identify variants that may be overrepresented in understudied populations. In 2021 three studies reported the genetic variants in the Latino population^13,19,20^, including that of Nuytemans et al. that identified novel *LRRK2* and *GBA* variants in Latino PD patients from the Caribbean^13^.

Our exploratory WES analysis identified four genes that carried variants with elevated AF in our patient group. COBL is a constrained gene (LOEUF 0.47, Table 2) related to actin cytoskeletal organization during neuronal morphogenesis and its expression is enriched in brain^21^. The role of EIF4G1 in the PD-related neurodegeneration is still debated^22^. TREM2, in contrast, has been confirmed to represent a candidate gene for PD susceptibility and progression, and soluble TREM2 (sTREM2) expression in cerebrospinal fluid was increased in PD patients, suggesting sTREM2 in CSF as a substitute immune biomarker for PD neuron injury^23^. CTSB belongs to the lysosomal proteases, and associations between the lysosomal pathway and PD pathogenesis has been described broadly. Lack of CTSB was shown to impair lysosomal trafficking during neural development^24^. However, because of our limited sample size, these results should be treated as a starting point for further studies in a much larger set of Latino PD cases.

While our study highlights the urgency to study genetics of these and other underrepresented populations, our WES included a small number of patients, therefore, we did not have statistical power to identify unique clinical differences or major clinical trends. Additional studies are needed, that not only evaluate known variants, but increase the WES/WGS data to identify population-specific risk variants in not only Puerto Rican, and Dominican, but also other Afro-Caribbean populations. This work will be aided by the Latin American Research Consortium on the Genetics of Parkinson’s Disease (LARGE-PD, Mata PI).

## Methods

### Participants

Evaluation of the frequency of LRRK2-p.G2019S and *GBA* variants was carried out in 151 participants in total: 32 with self-reported Puerto Rican ethnicity, and 119 with non-Hispanic-non-Jewish European ancestry from MSBI, SUNY Downstate and James J. Peters Veterans Affairs Medical Center (Bronx). Subsequently, 8 participants of Puerto Rican and 4 of Dominican ethnicity who were negative in the *LRRK2* or *GBA* testing, and who had higher likelihood of genetic etiology because of younger age of onset and/or positive FH, were selected for WES. We chose 57 years as cut-off for age of onset as this was the median age in our overall sample, and included patients with any FH, but did not limit to first-degree relatives. One Puerto Rican participant was subsequently excluded from the AF analyses due to discovery of biallelic *PRKN* deletions on an independent clinical testing including MLPA.

All participants met criteria for PD and signed informed consents from their respective institutions. The study was approved by and conforms with all Ethical Regulations of the Mount Sinai Internal Review Board and SUNY Downstate Internal Review Board.

### Genotyping for GBA variants

Genomic DNA (50–80 ng) was used to PCR amplify 5 fragments suitable for Sanger sequencing. Fragments were designed to include 10 common GBA variants (84GG, IVS2 + 1, p.E326K, p.T369M, p.N370S, p.V394L, p.D409G, p.L444P, p.A456P, and p.R496H). Primer sequences for PCR fragments were chosen to avoid amplification of the GBA pseudogene and are available upon request. Following PCR amplification, fragments were cleaned enzymatically with ExoSAP-IT (Affymetrix, Santa Clara, CA) according to manufacturer instructions. Samples were sequenced using ABI BigDye Terminator chemistry (v1.1) and visualized on an ABI 3730xl DNA analyzer. ABI sequencer files for each sample were compared to GBA consensus sequence to identify variants using Mutation Surveyor software (SoftGenetics, State College, PA). In addition to DNA sequencing, a PCR amplification assay was designed to assess presence of the RecNciI recombinant allele (primers and conditions available upon request).

### Evaluation of *LRRK2* and *GBA* variant frequencies

The LRRK2-p.G2019S and 11 variants in *GBA* (84GG, IVS2 + 1, p.E326K, p.T369M, p.N370S, p.V394L, p.D409G, p.L444P, p.A456P, RecNciI, and p.R496H) were analysed as described previously^25,26^ and Supplementary Methods]. Logistic regression models were used to evaluate the association between the self-reported ethnicity and the LRRK2-p.G2019S variant status, and the *GBA* variant status, respectively (STATA16, Texas). Two models were run: (i) with and (ii) without including “age of onset” as a co-variate. No difference in results were observed when adjusting vs not adjusting for this co-variate (data not shown). *T*-tests and Mann–Whitney tests were used to assess group differences.

### Whole exome sequencing

We performed WES on 12 samples (8 PR and 4 DR) to detect functional rare variants in 74 candidate genes (see Supplementary Methods for details on WES, Supplementary Table 2 for candidate genes), chosen based on a literature review for genes previously associated with PD and other movement disorders with phenotypic overlap including frontotemporal dementia (FTD), dystonia, essential tremor (ET), chorea-acanthocytosis (ChAc), McLeod Syndrome (MLS), Huntington disease-like 2 (HDL2), neurodegeneration with brain iron accumulation disorders (NBIA) and Alzheimer’s disease (AD). Additionally, we searched for loss-of-function mutations exome-wide. To test for the enrichment of more common variants in candidate genes in the PD population, Fisher’s tests were performed using allele counts in our WES cohort vs. published allele counts in a population database (gnomad v2).

WES was performed using the Agilent SureSelect Human All Exon V4 + UTR (71 Mb) library and an Illumina HiSeq 2000 with a paired-end module. Burrows–Wheeler Aligner (bwa-mem)^27^ was used to align reads to the reference sequence GRCh37/hg19, after which quality control, duplicate read removal, base quality recalibration, and variant calling was performed using Haplotypecaller in Genome Analysis ToolKit (GATK) version 3.3^28^. The mean per-base coverage for called variants ranged from 25-34X across samples. Single nucleotide variants and small in/dels were annotated with in silico predictions using the Variant Effect Predictor module from Ensembl (http://useast.ensembl.org/info/docs/tools/vep/index.html?redirect=no), in particular, the CADD (https://cadd.gs.washington.edu/), SIFT (https://sift.bii.a-star.edu.sg/), and Polyphen (http://genetics.bwh.harvard.edu/pph2/) scores, and via MACIE scoring^29^, (protein score > 0.80), which was eventually used to include variants of interest based on an estimate of the probability of a functional variant at the locus.

Stop and splice-acceptor/donor mutations detected in canonical transcripts genome-wide were filtered for quality using LOFTEE (Loss of Function Transcript Estimator) scores from the VEP (Variant Effect Predictor) plugin of Ensembl. As such, based on criteria set by the program, variants that are near the end of the transcript are filtered out^30^.

In testing for the enrichment of variants in the PD population, Fisher’s tests were limited to alleles found in >3 out of 11 individuals in our cohort, and those that had a higher variant alle frequency in PD (“risk alleles”). *p*-values were corrected for multiple testing using the false discovery rate method.

### Reporting summary

Further information on research design is available in the Nature Research Reporting Summary linked to this article.

### Supplementary information

Supplementary information

Reporting summary

## Data Availability

The IRBs of the participating institutions do not allow direct access to datasets used and/or analysed during the current study. Datasets are available directly from from Laurie Ozelius (laurie.ozelius@mgh.harvard.edu) or from Rachel Saunders-Pullman (Rachel.Saunders-Pullman@mountsinai.org) upon request with approval of a data use agreement.
