# Supplementary material for: Genetic risk variants in New Yorkers of Puerto Rican and Dominican Republic heritage with Parkinson’s disease
Source: NPJ Parkinsons Dis. 2023 Dec 7;9:160. doi: 10.1038/s41531-023-00599-6 (PMC10703927; doi:10.1038/s41531-023-00599-6)
Supplement: Supplementary file 1 — Supplementary information [file 41531_2023_599_MOESM1_ESM.pdf]

Supplementary Table 1. Variants in candidate genes that were found in >3 out of 11 unrelated individuals in our WES sample cohort. Comparison summary between allele frequency (AF) from PD and gnomAD database using the Fisher's exact test. Note: PD: Parkinson’s disease; AF: Allele frequency; NFE: European (non-Finnish); n.t.: not tested. Bold: nominally increased frequency of rs2230570 (*EIF4G1*) compared to the Latino population.

Gene Symbol	SNP	PD AF	All AF	p-value adjusted	Latino AF	p-value adjusted	African AF	p-value adjusted	NFE AF	p-value adjusted
DNAJC6	rs4915691	0.1818	0.1733	1.000	0.1635	1.000	0.4918	n.t.	0.03717	0.1552
EIF4G1	rs2230570	0.1364	0.02318	0.1573	**0.008691**	**0.05183**	0.08806	0.9580	0.02319	0.1311
LRRK2	rs7308720	0.2273	0.08740	0.2413	0.1616	0.9146	0.1416	0.6461	0.07153	0.1441
MAPT	rs2258689	0.2727	0.2762	n.t.	0.3772	n.t.	0.1474	0.4689	0.2098	0.9245
HS1BP3	rs2305458	0.2727	0.4834	n.t.	0.4019	n.t.	0.3264	n.t.	0.5729	n.t.
CTSB	rs12338	0.5000	0.3772	0.7407	0.3509	0.5668	0.3300	0.4532	0.3821	0.7174
SYNJ1	rs2254562	0.3182	0.2779	0.9352	0.2387	0.9183	0.2970	1.000	0.2739	0.9539
SMPD1	rs1050239	0.3636	0.1921	0.3118	0.1679	0.1560	0.1360	0.1796	0.2301	0.4824
LRRK2	rs11564148	0.2273	0.2965	n.t.	0.2065	1.000	0.1822	1.000	0.3068	n.t.
CDH23	rs4747195	0.3182	0.3054	>1.000	0.4278	n.t.	0.2475	0.9037	0.2679	1.000
CDH23	rs4747194	0.3182	0.3121	>1.000	0.4305	n.t.	0.2682	0.9992	0.2695	0.9739

PRNP	rs1799990	0.4091	0.3098	0.8845	0.4120	n.t.	0.3382	0.9555	0.3382	0.9249
SLC39A14	rs896378	0.4545	0.5307	n.t.	0.5020	n.t.	0.7342	n.t.	0.4754	n.t.
LRRK2	rs3761863	0.6364	0.6191	>1.000	0.4968	0.6216	0.5307	0.9004	0.6669	n.t.
EIF4G1	rs2178403	0.8182	0.7587	1.000	0.8233	n.t.	0.9555	n.t.	0.7452	1.000
COMT	rs4680	0.3182	0.4597	n.t.	0.4046	n.t.	0.3109	>1.000	0.5198	n.t.
ATXN3	rs1048755	0.2727	0.2677	>1.000	0.1550	0.4617	0.3476	n.t.	0.2417	1.000
PARK2	rs1801582	0.3182	0.1638	0.3582	0.1177	0.1525	0.1748	0.3933	0.1613	0.3778
ATXN2	rs695871	0.3182	0.6075	n.t.	0.5657	n.t.	0.3135	>1.000	0.7859	n.t.
NOTCH3	rs1044009	0.5909	0.7130	n.t.	0.5549	1.000	0.5354	0.9585	0.7899	n.t.
LRRK2	rs2256408	0.9545	0.9904	n.t.	0.9966	n.t.	0.8974	1.000	0.9999	n.t.
EIF4G1	rs13319149	1.000	0.9980	>1.000	0.9992	>1.000	0.9990	>1.000	0.9968	>1.000

Supplementary Table 2. Genes included in the candidate gene analysis. FTD fronto-temporal dementia; AD Alzheimer´s disease; NBIA Neurodegeneration with brain iron accumulation; DYT dystonia; ET essential tremor; ChAc Chorea-acanthocytosis (VPS13A disease); HDL Huntington disease-like syndrome; MLS McLeod syndrome.

Gene	Disease	Gene	Disease	Gene	Disease	Gene	Disease
ATP6AP2	PD/parkinsonism	*POLG*	PD/parkinsonism	*PRKAR1B*	PD/parkinsonism, FTD	*APOE*	AD
ATXN2	PD/parkinsonism	*PRKN*	PD/parkinsonism	*CHCHD10*	PD/parkinsonism, FTD, AD	*APP*	AD
ATXN3	PD/parkinsonism	*RAB29*	PD/parkinsonism	*BDNF*	PD/parkinsonism, AD	*BIN1*	AD
BST1	PD/parkinsonism	*SMPD1*	PD/parkinsonism	*ATP13A2*	PD/parkinsonism, NBIA	*CDH23*	AD
CHCHD2	PD/parkinsonism	*SNCA*	PD/parkinsonism	*PLA2G6*	PD/parkinsonism, NBIA	*CNTNAP5*	AD
CORO1C	PD/parkinsonism	*TMEM175*	PD/parkinsonism	*GABRA1*	DYT	*COMT*	AD
CTSB	PD/parkinsonism	*VAMP4*	PD/parkinsonism	*SLC1A2*	ET	*PSEN1*	AD, FTD
DNAJC6	PD/parkinsonism	*VPS35*	PD/parkinsonism	*FUS*	ET, FTD	*PSEN2*	AD
DNM3	PD/parkinsonism	*DNAJC12*	PD/parkinsonism/DYT	*C9orf72*	FTD	*TOMM40*	AD
EIF4G1	PD/parkinsonism	*GCH1*	PD/parkinsonism/DYT	*CHMP2B*	FTD	*VPS13A*	ChAc
FBXO7	PD/parkinsonism	*SLC30A10*	PD/parkinsonism/DYT	*GRN*	FTD	*PRNP*	HDL1, FTD, AD

GAK	PD/parkinsonism	*SLC39A14*	PD/parkinsonism/DYT	*ITM2B*	FTD	*JPH3*	HDL2
GBA	PD/parkinsonism	*SLC6A3*	PD/parkinsonism/DYT	*SQSTM1*	FTD	*XK*	MLS
HTRA2	PD/parkinsonism	*SYNJ1*	PD/parkinsonism/DYT	*TARDBP*	FTD	*C19orf12*	NBIA
LRRK2	PD/parkinsonism	*HS1BP3*	PD/parkinsonism, ET	*TBK1*	FTD	*FTL*	NBIA
MTX1	PD/parkinsonism	*LINGO1*	PD/parkinsonism, ET	*UBQLN2*	FTD	*PANK2*	NBIA
NOTCH3	PD/parkinsonism	*LINGO2*	PD/parkinsonism, ET	*CSF1R*	FTD, AD		
PARK7	PD/parkinsonism	*DCTN1*	PD/parkinsonism, FTD	*TREM2*	FTD, AD		
PINK1	PD/parkinsonism	*MAPT*	PD/parkinsonism, FTD	*VCP*	FTD, AD		

Supplementary Figure 1.

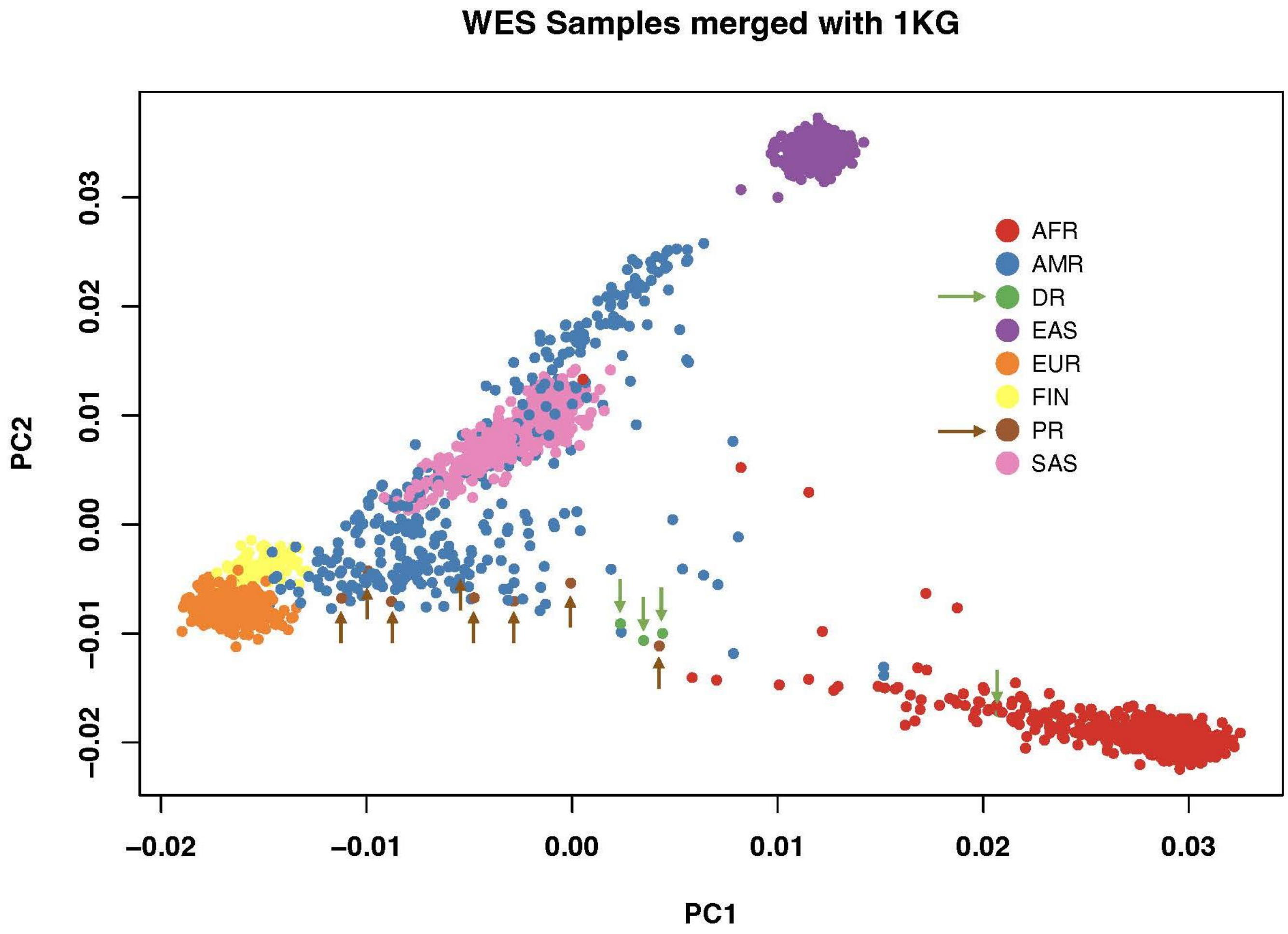

Principal component analysis for ancestry related markers for patients of Puerto Rican and Dominican ethnicity included in the WES analyses. All of the different 1KG (1000 Genomes) superpopulations are indicated. Note: PR Puerto Rican patients of our study (brown dots/arrows), DR Dominican patients of our study (green dots/arrows), AFR African, AMR American, EAS East Asian, EUR European, FIN Finnish, SAS South Asian.
